# Paleontology: advancing China's international leadership

**DOI:** 10.1093/nsr/nwy132

**Published:** 2018-11-12

**Authors:** Hepeng Jia

**Affiliations:** Writes for NSR from Beijing

## Abstract

In recent years, Chinese scientists have achieved significant progress in paleontological discoveries and scientific studies. Series of studies published in top journals, such as *Science*, *Nature* and *Proceedings of the National Academy of Sciences of the United States of America* (*PNAS*), have astonished the world by presenting beautiful fossils that furnish robust evidence to enrich the understanding of organismic evolution, major extinctions and stratigraphy. It has been portrayed as the heyday in the paleontology of China. What is the status of the field? What factors have caused the avalanche of fossil discoveries in China? What implications can these new discoveries provide for our understanding of current evolution theories? How, given their significant contribution to the world's paleontology scholarship, can Chinese scientists play a due leadership role in the field? At an online forum organized by the *National Science Review* (NSR), its associate editor-in-chief, Zhonghe Zhou, asked four scientists in the field as well as NSR executive editor-in-chief Mu-ming Poo to join the discussion.

Jin Meng

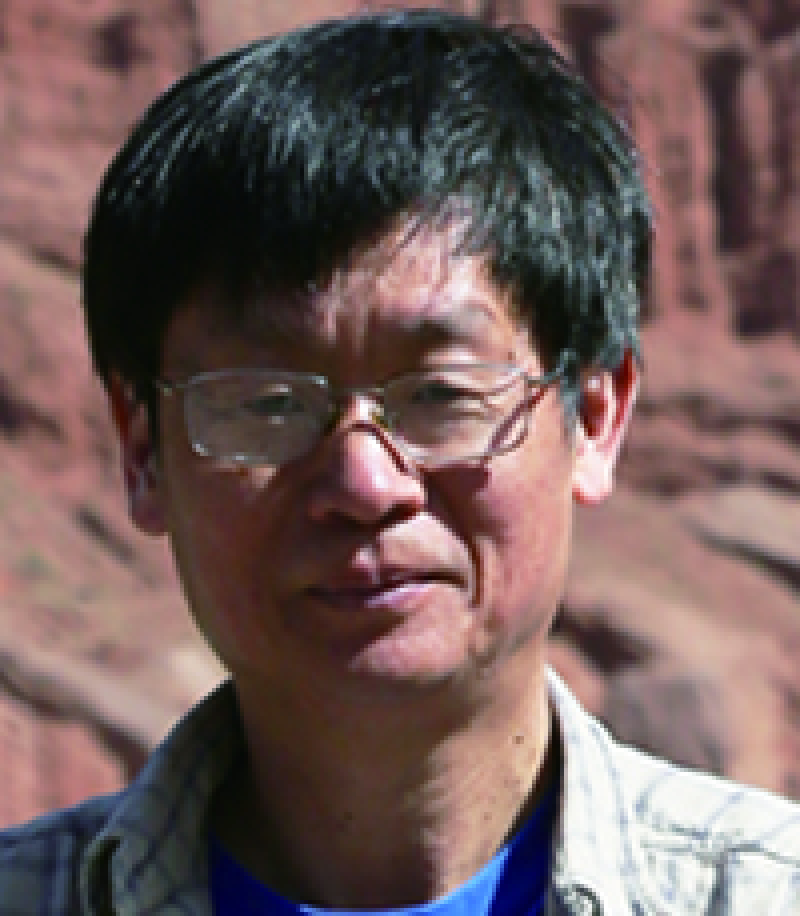

Paleobiologist at American Museum of Natural History

Mu-ming Poo

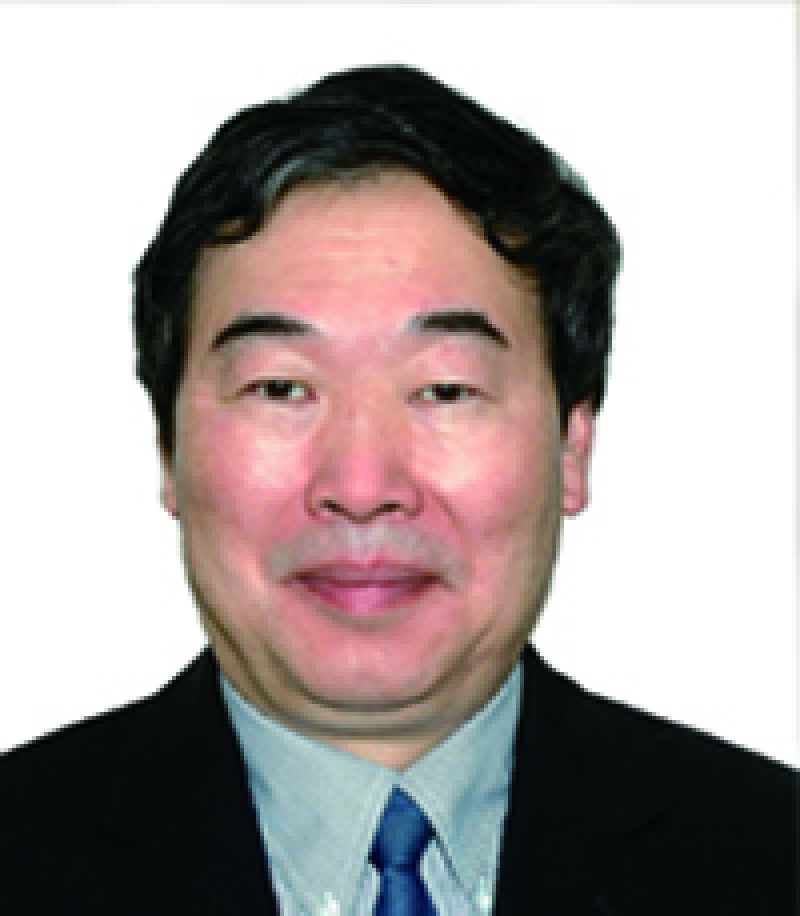

Neurobiologist at Center for Excellence in Brain Science and Intelligence Technology, Chinese Academy of Sciences

Shuzhong Shen

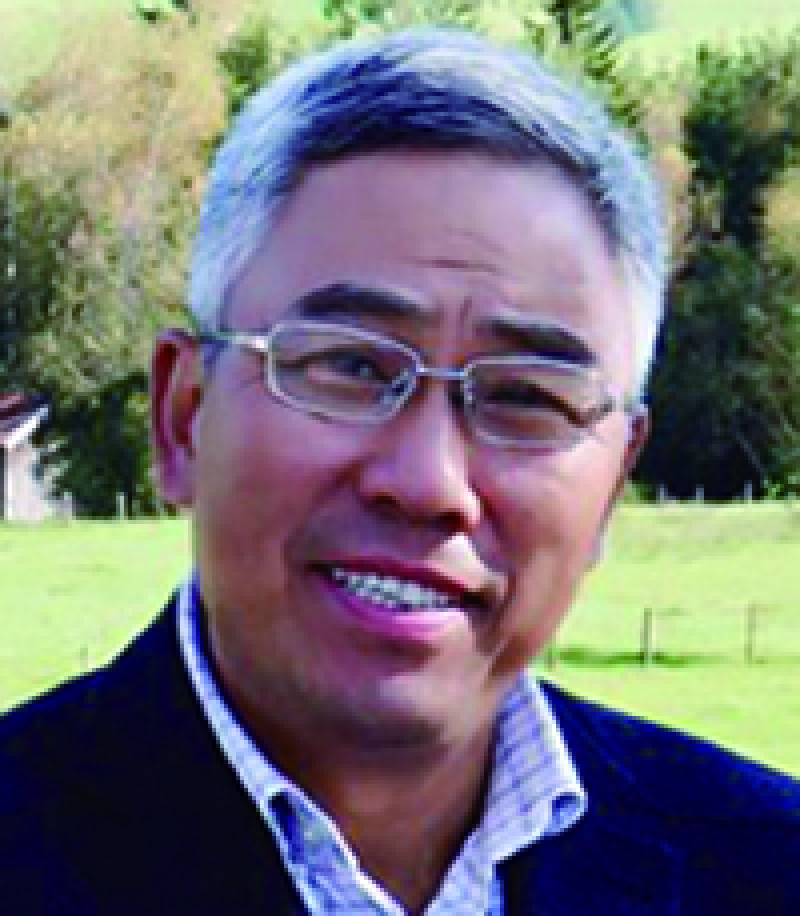

Stratigrapher at Nanjing Institute of Geology and Paleontology, Chinese Academy of Sciences

Shuhai Xiao

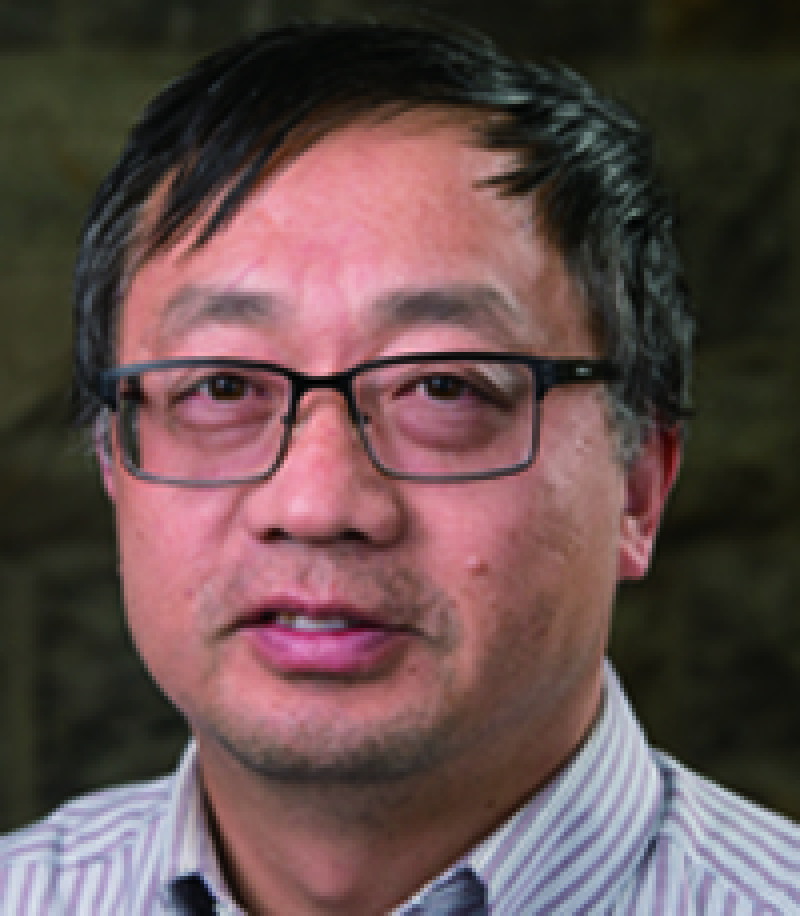

Paleobiologist and geobiologist at Virginia Polytechnic Institute and State University

Zhonghe Zhou (Chair)

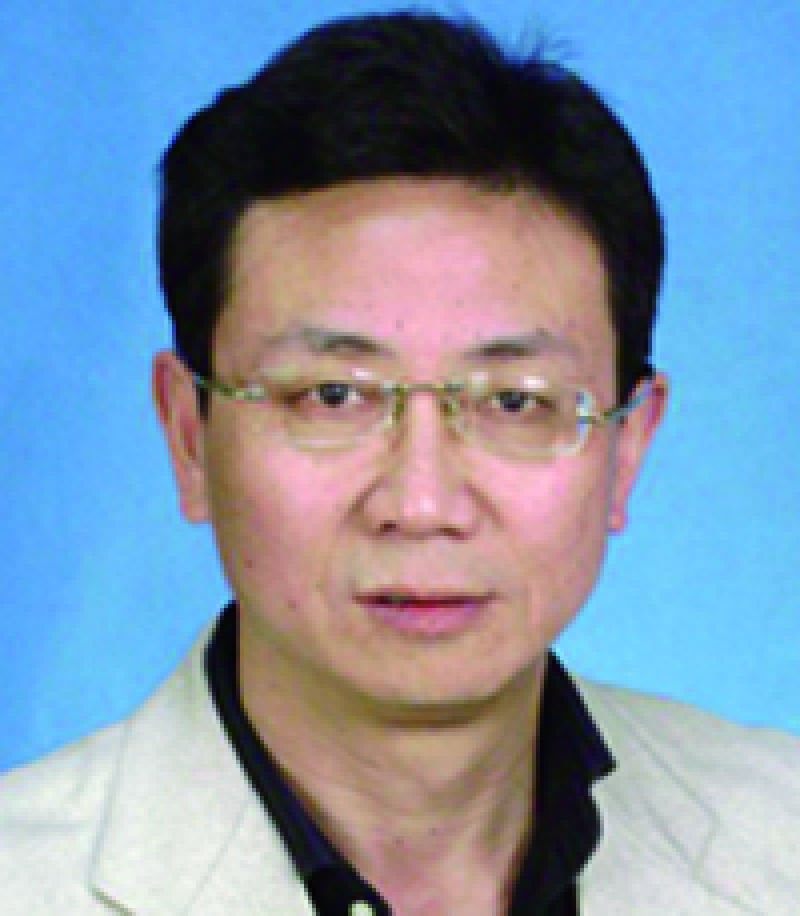

Paleobiologist at Institute of Vertebrate Paleontology and Paleoanthropology (IVPP), Chinese Academy of Sciences

## REMARKABLE ACHIEVEMENTS IN CHINA'S PALEONTOLOGICAL STUDIES

**Zhou:** In recent decades, Chinese paleontological research has achieved significant progress and success. Before we explore other aspects of paleontological studies, it is necessary to first sketch our major achievements. Perhaps Jin can go ahead first to summarize.

**Meng:** The most remarkable achievement is the huge amounts of paleontological fossils unearthed in China in different locations. Three major fossil geographical clusters of paleontological fossils—the Western Liaoning fossil sites (Jehol Biota) in Liaoning Province and its neighboring Hebei Province and Nei Mongol Autonomous Region, Weng'an fossil sites (Weng'an Biota) in Guizhou Province and Chengjiang fossil sites (Chengjiang Biota) in Yunnan Province—are the most famous ones. Findings in these three regions have filled many critical gaps in fossil records that are key to understanding evolutionary biology and largely changed our view about biodiversity in deep geological time. But in fact, China's paleontological findings are widely distributed, far beyond the three regions. For example, Xinjiang Uyghur Autonomous Region and Shanxi, Gansu, Shandong, Henan and Zhejiang provinces also witness excavations of many important paleontological fossils, and the fossil records range from Precambrian to the Cenozoic.

The most significant aspect of China's paleontological studies is the concentrated discovery of many important fossils in a short period of time. In the West, there have been up to 200 years' exploration of fossils. The findings left many gaps in evolution history. But in China, in two to three decades, the intensive excavations have rapidly revealed many stunning fossils of many organismic groups.

For example, the early Cambrian (editor's note: about 530 million years ago) Chengjiang Biota is represented by a diverse array of marine animal fossils with hard skeletal parts as well as soft parts preserved in detail. In the early Cretaceous Jehol Biota, discoveries of dinosaurs (including birds) with feathers and complete skeletons enlightened our understanding of the evolutionary transition from dinosaurs to birds; similar fossil preservations with great morphological evidence are also known from other groups, such as pterosaurs, mammals, insects and plants. These fossils not only help us to understand the morphologies of the organisms and their evolution, but also provide information on the ecosystem of the Earth in several critical time intervals during the geological history.

**Xiao:** Yes. The Chengjiang Biota is unique in its preservation of various species representing numerous animal phyla that have never been seen in older rocks. The geologically abrupt appearance of animal phyla has been popularly known as the Cambrian Explosion or Cambrian Radiation. The Chengjiang Biota enables us to understand animal evolution in greater details during

The most significant aspect of China's paleontological studies is the concentrated discovery of many important fossils in a short period of time.

—Jin Meng

the Cambrian Explosion. We can tell many stories about the evolution and development of early animals, including vertebrates.

**Meng:** The Late Triassic Guanling Biota in Guizhou is another example of landmark discovery. The Upper Triassic strata of around 227 million years ago preserved numerous marine fossils, featuring many intact and exquisite rare marine reptiles and sea lily (crinoids) fossils, accompanied by fish, bivalves and brachiopods. It is an important site to study the origination of several groups of vertebrates. One example is the study on a stem turtle (editor's note: a new taxon *Eorhynchochelys sinensis* that was published online on 22 August in *Nature*; the animal lived about 230 million years ago and studied by a team of scientists led by Chun Li from IVPP, Chinese Academy of Sciences); the new fossil provided the newest footnote for turtle evolution by showing how the reptiles developed features such as a beak and shell that are characteristic of the modern turtles.

**Shen:** What is significant is not only the number of fossils but also their completeness in certain geological records. Fossils are a window to deep geological history, but it is their hosting strata that endow meanings to them. First, they are used to establish the time frame in which fossils existed. Second, their correlation can also be used to judge the spatial distribution of natural resources. Third, their paleoecologic and morphological information reveal the environment, climate and other natural conditions these ancient lives lived with.

**Zhou:** Professor Shen, you are an expert in stratigraphy and mass extinctions. Could you please share your thoughts on the significant breakthroughs Chinese paleontological science has achieved in this aspect?

**Shen:** The fossils unearthed in China are not only of high quality but also demonstrated the continuation of different strata. This leads some significant Chinese fossil sites to be used to determine the world's chronostratigraphic standard. For example, the Meishan section in Changxing County of Zhejiang Province has been considered one of world's most important standards in determining geological era. Meishan site represents the transition from the Permian Period to Triassic Period. The Permian is the last period of the Paleozoic Era. The Triassic is the earliest period of the Mesozoic Era. So, it marks one of the most critical transitions during the Earth history.

Biostratigraphy studies geological time based on fossil records. Only by setting up a world's universal chronostratigraphy can we understand the evolution process of the Earth. Since 1960s, the International Commission on Stratigraphy (ICS) has adopted ‘one nail at a point' (Global Stratotype Section and Point, GSSP) strategy to determine chronostratigraphic boundaries. So far, among the 101 GSSPs, 11 have been established in China and the 12th from China will soon be ratified by the International Union of Geological Sciences (IUGS), which embodies significant contributions from Chinese stratigraphers. The two GSSPs at Meishan, Changxing are among the most important also due to their unique record to calibrate the largest biological mass extinction that occurred about 252 million years ago.

To become such a GSSP site, the excavated section must contain essentially continuous marine deposition, abundant fossils and distinct markers. The selected section should be well studied and collected and the results of the investigations published. Meanwhile, Chinese scientists who study these sections must offer easy accessibility and reasonable assurance of free study, collection and long-range preservation. Only after such requirements are achieved can scientists propose and finally determine such a chronostratigraphic standard point. Therefore, establishing 12 such GSSPs in a relatively short period of time is very significant, demonstrating high level of research capacity and leadership role of Chinese scientists in stratigraphy and paleontology.

The advantage of China in this aspect is embodied in that our fossils and stratigraphic sections can present very clear time slices. Environmental scientists also join our team to explore these fossil sites because they can tell us the impacts of environmental changes on past organisms and their living habitats. Numerous geochemical investigations also suggested that the mass extinctions are clearly a result of such environmental impacts. Among the big-five mass extinction events, fossil records in China can reveal at least the first three ones—the end-Ordovician Extinction, the late Devonian F/F Extinction and the end-Permian extinction—resulted from massive environmental changes.

**Zhou:** Then, how about the last two extinctions—the Triassic–Jurassic extinction and the Cretaceous–Paleogene extinction?

**Shen:** Evidence for the end-Triassic extinction is relatively weak in China because in the period, seawater withdrew from most parts of China, and relevant fossils in the continental deposits near the end-Triassic are relatively fewer. For the end-Cretaceous extinction, people's attention was overwhelmingly focused on asteroid impact, which is widely considered as the cause for the extinction of dinosaurs. The direct geological evidence of asteroid impact—such as the anomaly of iridium and impact quartz—has seldom been found in China, whereas they were widely reported in North America or Europe.

The end-Cretaceous mass extinction is perhaps the best-known extinction given its link to dinosaurs, effectively ending 76% of life on Earth 66 million years ago. For this extinction, we lack very accurate temporal link between the fossil record and the impact event. Personally, I think that the asteroid impact is

What is significant is not only the number of fossils but also their completeness in certain geological records.

—Shuzhong Shen

not the cause leading to the extinction. The asteroid impact occurred in very short period while the extinction lasted millions of years in view of the stratigraphic ranges of the fossils. In fact, the extinction pattern may not be so accurate, since we now know many dinosaurs became extinct before the impact event, and some of them were early birds evolving to modern birds.

## WHAT PROMOTES THE SIGNIFICANT ACHIEVEMENTS?

**Zhou:** Let's discuss factors underlying the fast progress of China's paleontology.

**Meng:** We are lucky to be working in a good era of discovery. The rapid economic development, mining activities and professional and amateur collections in China have all contributed to unearthing many fossils. Impressive funding sources, high-tech equipment, talent students and international collaborations have made all these practical. Utilization of these new findings have greatly advanced the paleontological research in China. In the past three or so decades since 1980s, fossil excavation and collection began to take off across China, ranging from Western Liaoning fossil sites (Jehol Biota) to Chengjiang Biota. Findings in the past three decades in Chengjiang Biota, for instance, have surpassed that of the Burgess Shale in Canada that has a collecting history over 100 years.

The models of fossil excavation and utilization in different Chinese regions are different. In Jehol Biota, amateur excavation and collection played a major role, whereas in the Chengjiang Biota, collections are more dominated by professional excavation. Sometimes, five or six teams of paleontologists race to work simultaneously.

**Xiao:** The fossil-containing rocks excavated by these teams are often up to several tons, retrieving a large number of fossils. Another important factor driving the rapid progress of paleontology in China is the broadened perspectives of Chinese researchers in the past 20 years. Many important fossils were discovered in the 1980s or earlier, but their significance was not realized until decades later. Extensive international exchanges since the 1980s helped Chinese scientists to better appreciate the scientific importance of China's fossil treasure and to place their fossils in a broader scientific context. For example, some of the most important fossils from the Weng'an, Chengjiang and Jehol biotas were described in Chinese journals in the 1980s or earlier, but it is in the most recent decades that their scientific significance was rediscovered and highlighted in international forums.

**Zhou:** Xiao's remarks supplement an important angle. China is a virgin land for discoveries of new fossils concerning many important questions regarding evolution of life in Earth history. Some of our newly achieved progresses are made on the fossils that had probably been sporadically unearthed during previous geological survey or agriculture activities, yet lacking immediate scientific recognition. With improved expertise and grant support, new excavations and studies can therefore make significant progress. In fact, the excavation in Jehol Biota began in 1920s. Why haven't we discovered a single specimen of now extremely abundant fossils, such as early birds and pterosaurs, over half a century? I believe they must have been unearthed, but we did not know them. With more systematic trainings in theories and skills, Chinese paleontologists now have developed expertise to deal with these findings.

**Meng:** Another advantage of China as a latecomer in paleontological research is that many geographic areas have remained untouched in fossil collecting and many research questions were unanswered and accumulated in previous studies. Any place you go and anything you find in the field are likely new. Paleontologists are overwhelmed with the massive findings in the past decades, and yet enjoyed the unearthed evidence that help to answer many scientific questions or provide clues to answer them. The aforementioned turtle study is one of them. The fossil's morphology answers how turtle developed its beak and shell in the evolutionary process, which has remained as a puzzling issue in previous studies on turtle evolution. Chinese paleontologists also achieved many important progresses in the studies on other vertebrate groups, such as primates and Mesozoic mammals.

## TOPPLING DARWINIST EVOLUTION?

**Zhou:** Xiao's remarks remind us the importance of evolutionary theories to guide paleontological findings and the latter's importance to enhance the theory. Because the intensive findings in Chengjiang has led to more use of the term Cambrian Explosion, some said this has challenged or even toppled the idea of gradual change in Darwin's evolution theories. How should we evaluate this or similar claims?

Personally, I don't think there is a big question. The central tenant of Darwin's evolution is not gradual change but natural selection, or heritable traits that help organisms survive nature's cruel conditions and become more common in a population over time. In Darwin's era, fossil preservation was not so complete, and in fact he had repeatedly argued the incomplete fossil record could be used to explain the ‘sudden appearance' of some major biological taxa such as many animal groups in the

The broadened perspectives of Chinese researchers drive the rapid progress of paleontology in China in the past 20 years.

—Shuhai Xiao

Cambrian and flowering plants in the Late Cretaceous. Some media have hyped the significance. In fact, the findings in Chengjiang and many other sites from the Cambrian or Precambrian ages exactly show evolution theory's correctness.

**Xiao:** To understand Darwin's claims, we should place them in a historical context. His idea of slow and gradual evolution was based on very limited paleontological data available to him at the time. The large amount of molecular and paleontological data available to us today allow us to tinker Darwin's theory, but it by no means overturns Darwin's central claim of evolution through natural selection. Such tinkering is not new, either. In fact, it has been suspected since the 1960s that animal evolution may have accelerated during the so-called Cambrian Explosion.

Even though ‘[Cambrian] Explosion' is a popular term in the scientific literature, it should be noted that such an ‘explosion' did not happen overnight. Animals emerged at least 600 million years ago and the Cambrian Explosion did not complete until 520 million years ago, and this is a period longer than the entire Cenozoic era (editor's note: from 66 million years ago when non-avian dinosaurs went extinct to the present time).

**Zhou:** In other words, the so-called ‘Cambrian Explosion' had occurred in tens of millions of years. In this sense, it is probably not an explosion at all. By the way, our panelist Professor Shen and his group have identified that end-Permian extinction occurred during a short geological time of around 31 000 years rather than the previously believed several million years. This will make the chronology of paleontological findings more accurate, and make the ‘life explosion' more unlikely. A more professional term should be ‘life radiation' rather than ‘life explosion'. Saying a [life] radiation is more accurate, indicating the period in question has witnessed the birth of more species than other comparable time.

**Meng:** Examining the relationship between evolution and the new fossil findings, we must contrast Darwin's era when there was no knowledge as evidence that we have today, such as molecular biology. When Darwin proposed the idea of natural selection, he used the analogy of artificial selection in domestic breeding of animals, which is a graduate process. But this time scale cannot be used in paleontology. Hence, we cannot say Darwin's evolution is featured with gradual change. In the early history of evolution, life appeared in different patterns at different time scales. At certain points of geological history, there were more radiations of newly appearing forms of life than other periods of time, but whether these are interpreted as radical changes or gradual change depend on how the changes are measured with which time scales.

**Shen:** The time scale of Darwin's investigation of evolution and that of geological eras are incomparable. The time unit of the earlier one is years or hundreds of years while that of the latter is million years. During the earliest 15 million years of the Cambrian Period, more than 20 phyla of lives appeared. This contrasts with that very few phyla occurred in the subsequent geological periods (around 500 million years).

**Meng:** It seems spectacular that in Chengjiang Biota, more than 20 phyla of animals suddenly appeared. But in terms of its significance, this is not a dramatic overhaul of evolution. Different geological eras have different features. The whole evolution history is a balance between points where life massively appearing and line where life was relatively more silent.

**Zhou:** The appearance of more than 20 phyla is indeed remarkable; however, it must be borne in mind that the rank of taxa is related to the time of the evolution of the lineage. If you look at the life radiation of the whole Cenozoic, you may not be able to recognize a new phylum or even a class, however there were more new orders or families of animals and plants. In my opinion, they seem more like an explosive radiation. If looking at more fossil sites, one can find a more balanced picture of life evolution. In China, important fossil sites are not limited to Chengjiang, Weng'an and Jehol biotas. In some cases, exceptionally preserved fossils such as organs and soft tissues that are normally hard to preserve can provide us many fresh new information about evolution.

**Meng:** Yes. The organ evolution of animals provides many examples of gradual change in evolution, such as the evolution of the mammalian middle ear. Many newly identified fossils evidenced animal species evolved in accordance with the prediction of [evolutionary] theories, further confirming evolution.

**Shen:** In the studies of dinosaur's evolution to birds, China's fossils have changed the world's understanding. It was previously recorded that earliest known bird fossils occurred earlier than the bird-like dinosaurs, which appears to be contrary to the theory of dinosaurian origin of birds. Now with dozens of new fossils from China, paleontologists can show a clear evolutionary lineage from dinosaurs to birds. These findings enriched our understanding of evolution rather than denying it.

## INTERNATIONAL COLLABORATION ON THE RISE

**Zhou:** As we have mentioned just now, China's tremendous growth in paleontology cannot be separated from active international collaboration. However, it is also found that many important publications in the field were co-authored by Chinese and foreign scientists who often positioned some important authorship role. How should we comment this situation?

The central tenant of Darwin's evolution is not gradual change but natural selection, or heritable traits that help organisms survive nature's cruel conditions.

—Zhonghe Zhou

In terms of international influence, Chinese paleontologists have done much better than most other disciplines.

—Mu-ming Poo

**Meng:** [International] collaboration is needed. In the past years, as we already mentioned, many fossils were unearthed; it is overwhelming, and many of the fossils have not been sufficiently studied. Hence, international collaboration is helpful and necessary. It can be expected, though, 10 or more years later, more and more Chinese scientists will become leading authors of their researches published in major international journals.

**Shen:** Like other scientific areas, China's paleontological research is from mimicry and following to participation, and now in some areas, Chinese scientists have played the leadership role. Many Chinese paleontologists are constrained in their English communication skills and background knowledge. They can give an accurate analysis of the material they have, but need international collaborators for the theoretical interpretation, development and application of the finding.

**Poo:** You paleontologists are very reserved. It seems to me that in terms of international influence, Chinese paleontologists have done much better than most other disciplines. Right now, there are many international mega science projects. In physics and astronomy, we mainly participated in others' projects. Can China initiate an international mega science project in paleontology?

**Shen:** The Deep Time Digital Earth (DDE) project being proposed may become a candidate mega science project. It plans to digitalize various geologic data including stratigraphy, paleontology, sedimentology, paleogeography, geochemistry, etc. Several international science organizations are advancing the project. IUGS plans to convene a scientific workshop in Xiangshan, Beijing to discuss this proposal. Its implementation needs large amount of data and is easier said than done. One ongoing project in the field that may be called a mega science project is the Geobiodiversity Database (GBDB) implemented by the Nanjing Institute (of Geology and Paleontology).

**Meng:** One example is the program ‘Assembling the Tree of Life (ATOL)' run by National Science Foundation (NSF, USA), which intended to explore the diversity and phylogeny of life on Earth through a multidisciplinary effort. After it was launched in mid-1990s, the ambitious program has been invested an impressive amount of funding. While significant results were generated from the effort, eventually it wound up hastily. To organize such diversified mega projects has always been challenging. For paleontology and geology, there is no political border that delimits the natural phenomenon and scientific problems. Perhaps, this kind of research can be integrated into Belt and Silk Initiative, in which Chinese scientists can play a leadership role, at least in Asia. In terms of building up sophisticate databases, China still lacks a group of talents who are capable and willing to bridge paleontological knowledge and data with computer science and database programing. This will be a long-term and time-consuming undertaking, and one may not expect to produce any SCI paper or harvest any quick result.

**Zhou:** I am relatively conservative towards mega science in paleontology. In most times, paleontology is featured with small team work. Mega projects here are mainly embodied in big data support. If a China-led mega project in paleontology is to succeed, there are several preconditions. First, Chinese paleontologists need to have a high academic status. Second, China must support the best research. Third, good data.

**Xiao:** A good research project, be it a mega project or not, should be driven by well-defined scientific questions. There have already been a few big paleontological projects in China, each with a budget of over tens of millions of yuan. Some of these projects were designed to address specific scientific questions. If the principle of scientific question-driven research continues to be pursued, it is anticipated that Chinese paleontologists will play a greater leadership role in international collaborations.

## CONSERVATION AND DEVELOPMENT

**Zhou:** Let's shift to the last topic of our forum today. As discussed above, China's paleontological development relies on fast economic development and amateur fossil explorers. While bringing many valuable fossils, these activities may also lead to the loss of geological and paleoenvironmental information. How should we deal with the tension?

**Meng:** Honestly speaking, much of our fossil discovery has mainly relied on the public. If only exploring fossils with their own hands, paleontologists may not see many invaluable fossils in their lifetime. Nonprofessional fossil hunting, however, has many disadvantages, including fabrication and losing geological information. As paleontologists, we must be very cautious in our research and try to track down geological and stratigraphic information of the collected fossils as completely and truthfully as possible.

**Zhou:** Grassroots fossil exploration is a double-bladed sword. To exert its advantage and curb its disadvantage, the basic solution relies on management of fossil collecting and protected sites. The current situation is caused by a lack of appropriate scientific management. Some local communities think exploring fossils can lead to a museum to attract tourists and lack incentives to hand out their fossils to paleontologists, or even they hand out their fossils, they have no awareness to preserve excavation site efficiently. The low scientific literacy of fossil collectors is another problem.

**Meng:** Development of tourism has impacted paleontological research. Some fossil sites were encircled to become tourism sites and sell tickets as a source of revenue. In some cases, scientists have lost their normal access to the fossil sites. Some tourism facilities have unfortunately chained or even destroyed the original geological landscapes. The economic activities are understandable, but we as scientists need to further popularize our science and educate the publics to call their attention to conservation of the fossil sites.

**Xiao:** In many cases, the excavation and discovery of new fossils was facilitated by mining activities. The Chengjiang and Weng'an biotas are both such examples. But excessive and poorly planned mining activities are destructive to paleontological resources. The site of Weng'an Biota, for example, has been almost destroyed by phosphorite mining. The Chengjiang Biota, on the other hand, is better protected, partly because of its designation as a UNESCO World Heritage Site. In such cases, well-planned tourism can be a good thing because it raises public awareness of the scientific importance of fossils and can also generate income to offset the losses from suspended mining activities. Such tourism and protection should be implemented so as not to compromise paleontological research.

**Shen:** Tourism based on paleontological findings has become a trend. Scientists have few choices but to collaborate with local communities and governments to promote their fossil resources. This can also win their supports for our research.

**Meng:** While regulation and management are important to ensure the positive side of economic activities and amateur collection, high-tech methods should be widely adopted to improve the accuracy of paleontological research. Meanwhile, interdisciplinary studies to bridge paleontology with climate change, biology and environmental studies should be encouraged.

**Zhou:** Today we have very good discussions. I think in the future we can further discuss some points raised today to advance our discipline.

**Figure 1:**
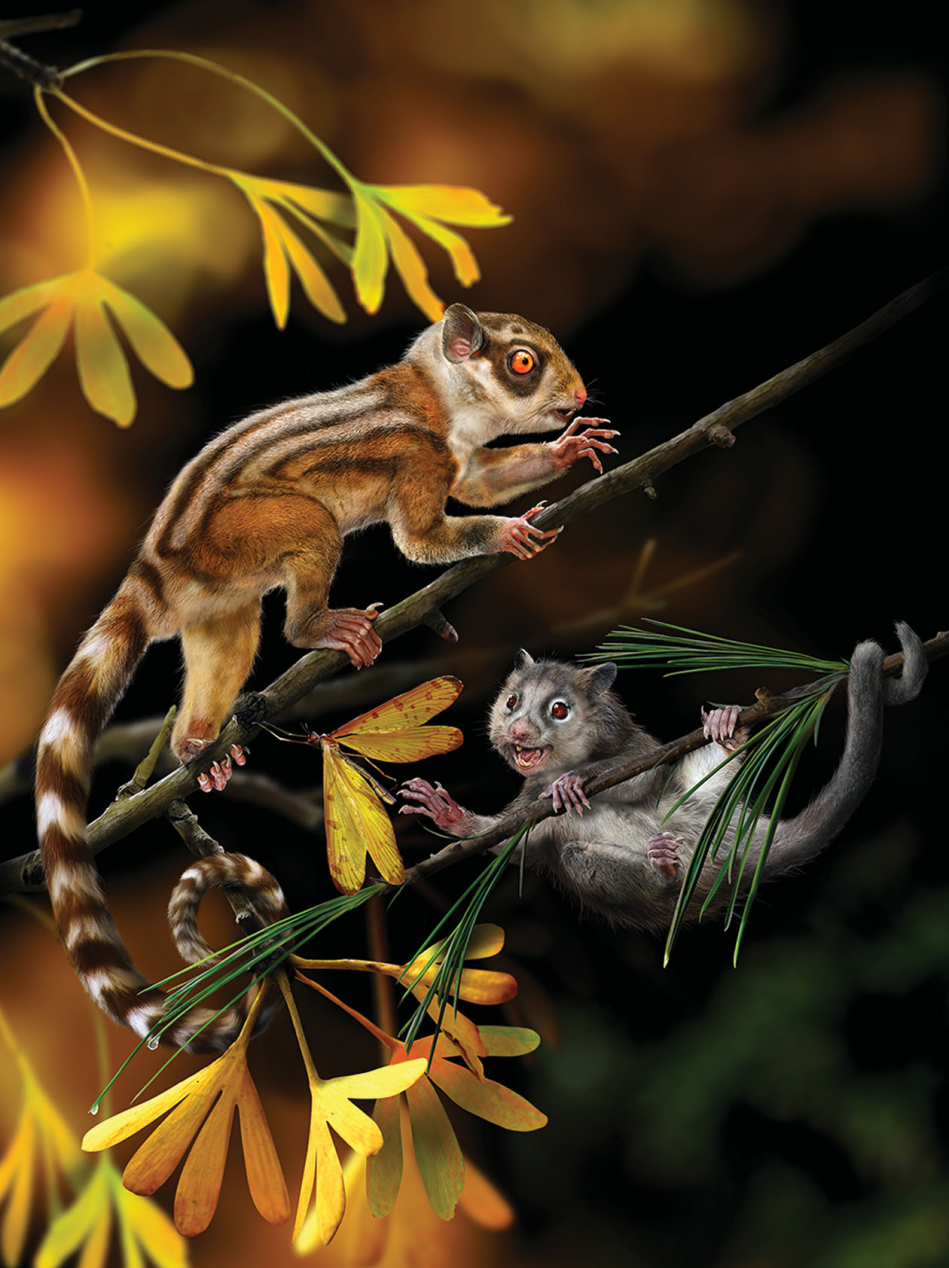
Reconstruction of the arboreal *Shenshou* and *Xianshou* from the extinct mammal group of Euharamiyida that had lived in the Jurassic forests. The fossils were found in north-eastern China (*Image credit: Chuang Zhao*).

**Figure 2:**
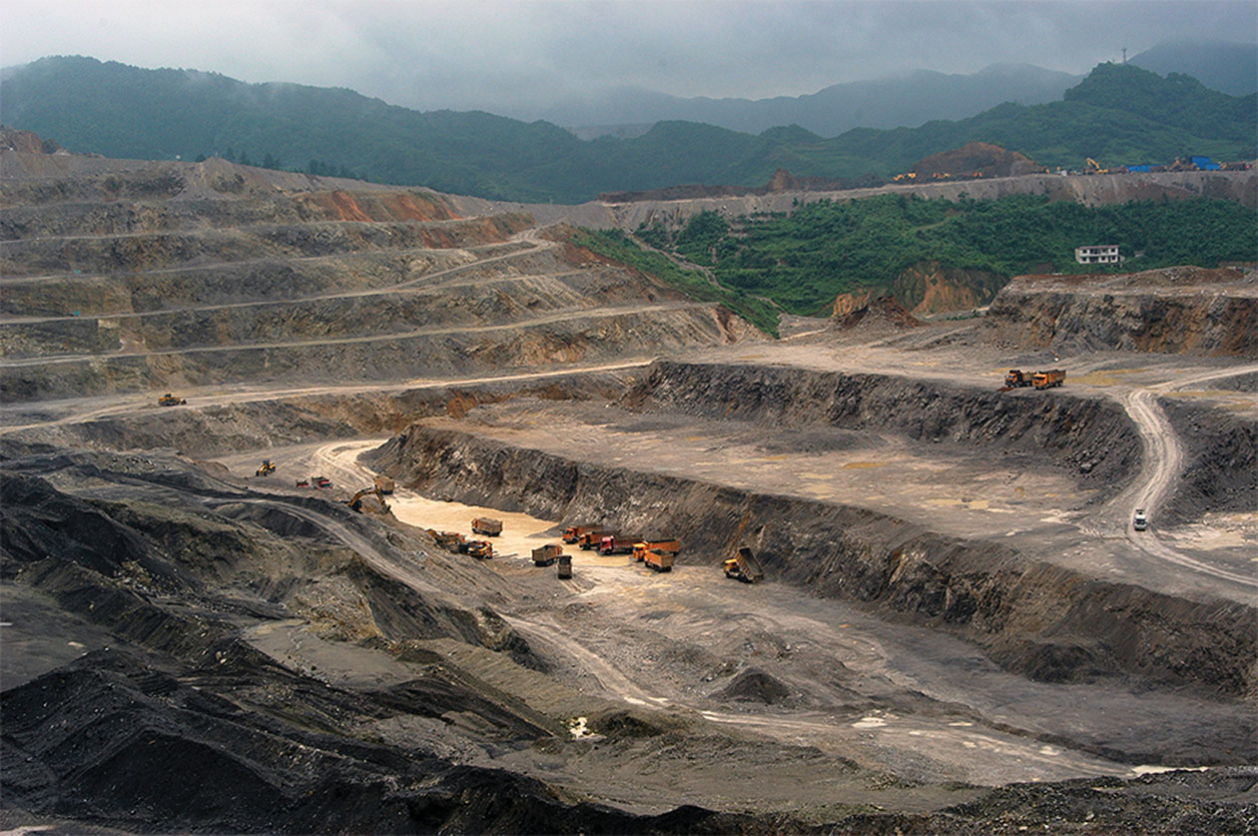
An important Precambrian fossil site turned into one of the largest phosphate mines in China (*Courtesy of Shuhai Xiao*).

